# Feature importance for estimating rating of perceived exertion from cardiorespiratory signals using machine learning

**DOI:** 10.3389/fspor.2024.1448243

**Published:** 2024-09-24

**Authors:** Runbei Cheng, Phoebe Haste, Elyse Levens, Jeroen Bergmann

**Affiliations:** ^1^ Natural Interaction Lab, Institute of Biomedical Engineering, Department of Engineering Science, University of Oxford, Oxford, United Kingdom; ^2^ Department of Technology and Innovation, University of Southern Denmark, Odense, Denmark

**Keywords:** fatigue, heart rate, breathing, ventilation, machine learning, physical activity, Yo-Yo test

## Abstract

**Introduction:**

The purpose of this study is to investigate the importance of respiratory features, relative to heart rate (HR), when estimating rating of perceived exertion (RPE) using machine learning models.

**Methods:**

A total of 20 participants aged 18 to 43 were recruited to carry out Yo-Yo level-1 intermittent recovery tests, while wearing a COSMED K5 portable metabolic machine. RPE information was collected throughout the Yo-Yo test for each participant. Three regression models (linear, random forest, and a multi-layer perceptron) were tested with 8 training features (HR, minute ventilation (VE), respiratory frequency (Rf), volume of oxygen consumed (VO2), age, gender, weight, and height).

**Results:**

Using a leave-one-subject-out cross validation, the random forest model was found to be the most accurate, with a root mean square error of 1.849, and a mean absolute error of 1.461 ± 1.133. Feature importance was estimated via permutation feature importance, and VE was found to be the most important for all three models followed by HR.

**Discussion:**

Future works that aim to estimate RPE using wearable sensors should therefore consider using a combination of cardiovascular and respiratory data.

## 1 Introduction

In sports, fatigue is commonly defined as “sensations of tiredness and associated decrements in muscular performance and function” and is a complex phenomenon that encompasses both physiological and psychological factors (1). The build-up of fatigue is considered a risk factor for injuries in sports, and prescribing sufficient training regimen to produce positive training outcome while minimising fatigue-related risk of illness and injury is a challenge constantly faced by coaches, sport scientists and medical personnel alike (2–5). Various monitoring techniques have been devised by sport scientists to capture exercise workloads as ways to address these issues, which can be further divided into two categories; (i) external workloads which are measures of the physical tasks performed by athletes, often captured via local/global positioning systems, inertial sensors, and camera-based player tracking systems; and (ii) internal workloads which are the physiological and psychological responses an athlete has towards the external workloads, most prominently captured via heart rate (HR) and ratings of perceived exertion (RPE) (2, 5, 6).

RPE is one of the most commonly applied and investigated internal workload measures in team sports, thanks to its intuitiveness and ease of use, as well as its ability to capture both physiological and psychological factors that impact exertion (5, 6). There are many variations of RPE scales developed over the years, but they all work on the same principle, consisting of a numerical scale with verbal descriptor corresponding to different levels of exertion. The two most popular RPE scales are the Borg 6-20 Category Scale and the Borg Category-Ratio-10 (CR-10) Scale (6, 7). One major shortcoming of RPE is that it is not very suitable for continuous monitoring of training and match workloads, since RPE is captured via active feed-back provided by the monitored athletes. Rather, RPE is often captured as a one-off measurement after a training session or match in the form of session RPE that aims to capture the overall exertion level of the session.

In terms of continuous on-field monitoring of athletes, the most commonly captured physiological measurements consist of heart rate (HR) and various HR-derived metrics, partly thanks to a high level of accessibility to commercially available validated sensor systems (5, 6). HR-based metrics are often more difficult to interpret than RPE and require greater physiological expertise to analyse (8). In pursuit of a continuous monitoring metric which is easy to interpret, researchers have been attempting to estimate RPE using physiological measurements that can be continuously monitored via sensor systems (9–15).

Much of the existing literature on estimating RPE using sensor systems leverages HR-based measures and movement data (9–12, 14, 15). RPE has been theorized to be dependent on both cardiovascular and respiratory factors (6), and several studies highlight the strong correlation between respiratory measures and RPE (13, 16). As such, this study aims to investigate the importance of respiratory measures in comparison to HR for estimating RPE using machine learning methods.

## 2 Materials and methods

### 2.1 Participants

This study was conducted with the approval of the Research Ethics Committee of the University of Oxford (R43470/RE001). For the study, 20 participants, between the ages of 18 to 43, were recruited. The volunteers were all well-informed on the purpose of the study and have given written consent to be included in the publications resulting from this work. All participants included were self-declared to be healthy and in shape to carry out the physical activities detailed in the experimental protocol. All participants resided in the UK and were proficient in English. Basic information about each participant was collected at the beginning of each data capture session, which includes age, sex, and self-reported weight and height. Of the 20 participants, 10 were female and between the ages of 19 to 40, with a weight distribution ranging between 50 kg and 87 kg, and a height distribution ranging between 150 cm and 182 cm. The other 10 participants were male and between the ages of 18 to 43, with a weight distribution ranging between 63 kg and 92 kg, and a height distribution ranging between 170 cm and 194 cm. The participants were invited to two identical data capture sessions scheduled weeks apart. This was done to capture variability within each participant and minimize the potential bias due to environmental factors, as well as to generate more training data per participant. Out of the 20 participants, 3 had one of their runs omitted due to equipment failure on the day of the data capture, and 1 was unable to attend a second session due to scheduling conflicts. Table 2 shows the max Yo-Yo test level and max RPE achieved by the participants in each data capture session.

### 2.2 Experimental setup

In each data capture session, the participant performed a Yo-Yo intermittent recovery level 1 (Yo-Yo IR1) test (17) on a hard-surface outdoor multipurpose sports court. The test consisted of bouts of progressive speed shuttle-running around a 20 m track with 10 s of active recovery, in the form of a short walk or jog around a 5 m track behind the start line, in between. The running speed was paced via audio cues, and the test terminated when two consecutive cues have been missed. The Yo-Yo IR1 test was chosen for this experiment for its ability to simulate workloads in sports characterized by intermittent high-intensity efforts, such as football, rugby, basketball and similar (18).

A modified version of Borg’s Category Ratio 10 scale (CR10) (13, 19, 20) was utilized to rate perceived exertion (RPE), see Table 1. Each participant was given an explanation of the RPE scale and time to familiarize themselves with it at the start of each session. The RPE level of the participant was recorded at the start and finish of each shuttle of the Yo-Yo test. To achieve this, the modified CR10 RPE scale along with the anchor words corresponding to the RPE level were written at the shuttle run track (0 m line), and the participant was asked to step on the writing that represents their RPE level at the time. This was both recorded by a researcher at the time as well as recorded via a camera stationed at the 0 m line.

**Table 1:** Modified RPE scale used in this study.

Rating	Descriptor
0	Rest
1	Very Easy
2	Easy
3	Moderate
4	Somewhat Strong
5	Strong
6	*
7	Very Strong
8	*
9	*
10	Extremely Strong

The participants were shown the scale before each data capture session. The scale along with the descriptors was also written in chalk on the 0 m line of the running track. During the Yo-Yo IR1 tests, the participants were instructed to indicate their RPE level by stepping on the corresponding number each time they crossed the 0 m line.

Each participant wore a COSMED K5 (COSMED, Rome, Italy) portable metabolic machine, and a Polar H10 (Polar Electro Oy, Kempele, Finland) heart rate monitor throughout the test, enabling the capture of respiratory frequency (Rf), minute ventilation (VE), volume of oxygen consumed (VO2) and heart rate (HR). The COSMED K5 has been validated against the state-of-the-art stationary metabolic machine and demonstrated reliable accuracy (21). The Polar H1 heart rate monitor has been validated against electrocardiography and shown to have acceptable reliability during exercise (22). In this experiment the Polar H10 was linked to the COSMED K5 via Bluetooth, and the COSMED K5 was set to breath-by-breath mode for data collection.

**Figure 1:**
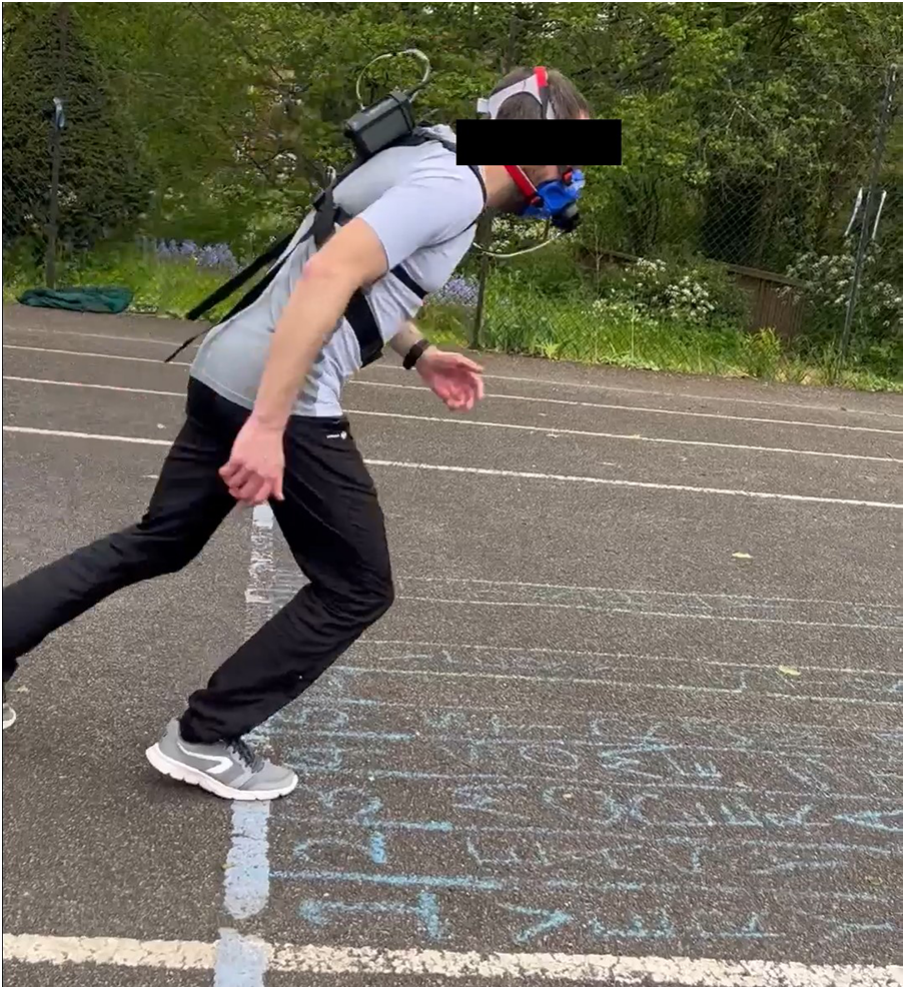
This figure shows a participant, wearing the COSMED K5 and a Polar H10 heart rate strap, at the starting (0 m) line at the beginning of a Yo-Yo test level. The Borg CR10 RPE scale, written in chalk, was marked in front of the start (0 m) line. The participant can be seen stepping on the number 3, indicating their RPE level was 3 at that instant.

Figure 1 shows a snapshot of the experimental setup at the 0 m line of the Yo-Yo test.

### 2.3 Data preprocessing

The RPE and COSMED data were recorded as time series spreadsheets and synchronized via the COSMED K5’s built-in GPS which allowed for the identification of the starting point of the Yo-Yo test. In the HR data, null values were present, likely due to motion artifacts. This artifact affects around 3% of the data, all impacted data containing a null value heart rate were omitted during pre-processing. The data was then divided into 10-s clips with no overlaps, and the average Rf, VE, VO2, HR, and RPE were obtained from these clips. The average Rf, VE, VO2, and HR values were then grouped with the participants’ physical characteristics: height, weight, gender, and age, as a set of features that were available to estimate the RPE.

### 2.4 Machine learning model selection

Three regression models were tested using this set of 8 features, a linear regression (LR) model, a random forest regression model (RFR), and a multi-layer perceptron (MLP) regression model. Both the linear model and the RFR model were built using the scikit-learn python package (23). The MLP was built via the Tensorflow library in Python3 (24) and consists of two dense layers with ReLU activation, and a regression layer that is a single fully connected node with a linear activation function. The hyperparameters of each model were tuned and the generalizability of each model was tested using leave-one-subject-out cross-validation (LOSOCV). In LOSOCV, each cross-validation iteration involved training the model on data from all but one participant which is then used to validate the model (25). The models were evaluated on root-mean-squared-error (RMSE) and mean-absolute-error (MAE). The results of the LOSOCV were used to compare the performance of the tuned models. Finally, using the optimized hyperparameters, each model was retrained using all the available data for the feature importance estimation.

### 2.5 Feature importance

Permutation feature importance (PFI) is a model inspection technique used to evaluate the significance of individual features to the prediction accuracy of a model (26). PFI works by evaluating a trained model using randomly shuffled values for each feature, simulating scenarios where each feature, in turn, provides no information to the model, thus measuring the impact of each feature on the accuracy of the model. Unlike parameter-based importance which can only be applied to certain models (such as impurity-based feature importance for RFR), permutation feature importance is model agnostic and can be applied to any model. In this study, PFI was used to inspect the importance of Rf, VE, VO2, HR, height, weight, gender, and age when estimating RPE using Linear, RFR, and MLP models.

## 3 Results

The maximum CR-10 RPE reached across the participants ranged from 7 to 10, with 14 participants reaching level 10, every participant’s max RPE level is detailed in Table 2.

**Table 2:** Results from the LOSOCV, N stands for the number of 10-s clips from each participant.

Metadata						Linear		RFR		MLP	
Participant	R1 Yo-Yo	R1 RPE	R2 Yo-Yo	R2 RPE	N	RMSE	MAE	RMSE	MAE	RMSE	MAE
D01	12.2	10	12.2	9	17	1.774	1.509	1.143	0.924	1.468	1.255
D02	14.2	10	14.3	7	50	1.595	1.266	1.137	1.004	1.618	1.142
D03	13.4	10	14.3	10	32	2.377	2.103	2.211	1.903	2.570	2.229
D04	14.2	8	15.1	9	64	1.312	1.155	1.449	1.266	1.433	1.211
D05	NA	NA	17.5	10	73	0.785	0.653	1.020	0.848	1.089	0.950
D06	17.8	10	19.4	10	177	3.403	3.260	2.047	1.692	2.245	1.929
D07	14.3	7	NA	NA	26	3.199	3.107	1.073	0.917	0.862	0.729
D08	18.1	10	17.5	10	155	2.335	1.698	1.899	1.484	1.778	1.353
D10	14.2	10	14.2	9	67	1.394	1.112	1.243	0.918	2.404	2.142
D11	NA	NA	20.1	10	108	3.109	2.736	1.839	1.601	1.603	1.213
D13	13.2	10	13.4	10	23	0.998	0.861	1.607	1.255	1.739	1.374
D14	13.2	10	13.2	10	32	3.501	3.049	3.555	2.988	3.310	2.812
D15	13.1	8	13.4	9	37	1.599	1.413	1.433	1.080	1.441	1.168
D16	15.8	9	16.7	10	111	1.370	1.204	1.948	1.519	1.353	1.155
D17	13.3	7	14.1	10	42	2.136	1.635	2.277	1.948	2.836	2.535
D18	12.3	9	13.2	9	30	1.181	0.883	1.290	0.924	1.596	1.395
D19	13.1	8	NA	NA	15	1.174	1.012	0.867	0.735	1.752	1.559
D20	16.2	10	16.2	10	106	1.750	1.476	2.086	1.713	1.898	1.642
D21	11.2	8	12.3	10	18	0.922	0.751	1.423	1.066	1.107	0.807
D24	11.2	8	12.1	8	24	0.924	0.758	2.367	2.152	1.431	1.204
All					1,207	**2.263**	**1.798**	**1.849**	**1.461**	**1.882**	**1.511**

Columns “R1 Yo-Yo” and “R2 Yo-Yo” show the max Yo-Yo text level achieved by the participant in the first round (R1) and the second round (R2) of the data capture, while columns “R1 RPE” and “R2 RPE” show the max RPE level achieved by the participant in each of these data capture rounds. Each row of the table shows the results of the cross-validation iteration where the data from the stated participant was selected as the validation set, thus left out of the training set. The last row shows the average performance of each model across all subjects. The RFR model (highlighted in green) has been shown to have the best performance overall, followed by the MLP model (highlighted in yellow, and finally the linear model (highlighted in red) in last place.

### 3.1 Model validation

For the RFR model tuning, the number of estimators was tuned between 50 to 200 at an increment of 50, and 100 estimators were found to be the most optimal. For the MLP model, the number of dense layer units (32, 64, 128), the learning rate (1e−3, 1e−4, 1e−5), and the epoch number (5–20) were tuned, and the final model was built with 128 units per dense layer, trained at 1e−3 learning rate for 15 epochs.

Table 2 shows the detailed test results of the leave-one-subject-out cross-validation. The RFR model is the most accurate overall, with a RMSE of 1.849 and MAE of 1.461±1.133 when averaging across all subjects. The RFR model was the second most consistent model across all subjects in the cross-validation, with a median of 1.528 and an interquartile range of 0.874 in RMSE, and a median of 1.261 and an interquartile range of 0.779 in MAE.

The MLP model placed second in overall accuracy but was found to have performed the most consistently in cross-validation, with a RMSE of 1.882 and MAE of 1.511±1.123 when averaging across all subjects; in LOSOCV, the MLP model had a median of 1.611 with an interquartile range of 0.640 in RMSE, and a median of 1.304 and an interquartile range of 0.624 in MAE.

The linear model performed the worst out of the three, with the highest RMSE (2.263) and MAE (1.798±1.374) when averaging across all subjects. Furthermore, the linear model was the most inconsistent in the cross-validation, with a median of 1.597 and an interquartile range of 1.1785 in RMSE, and a median of 1.340 and an interquartile range of 0.953 in MAE.

### 3.2 Feature importance

**Figure 2:**
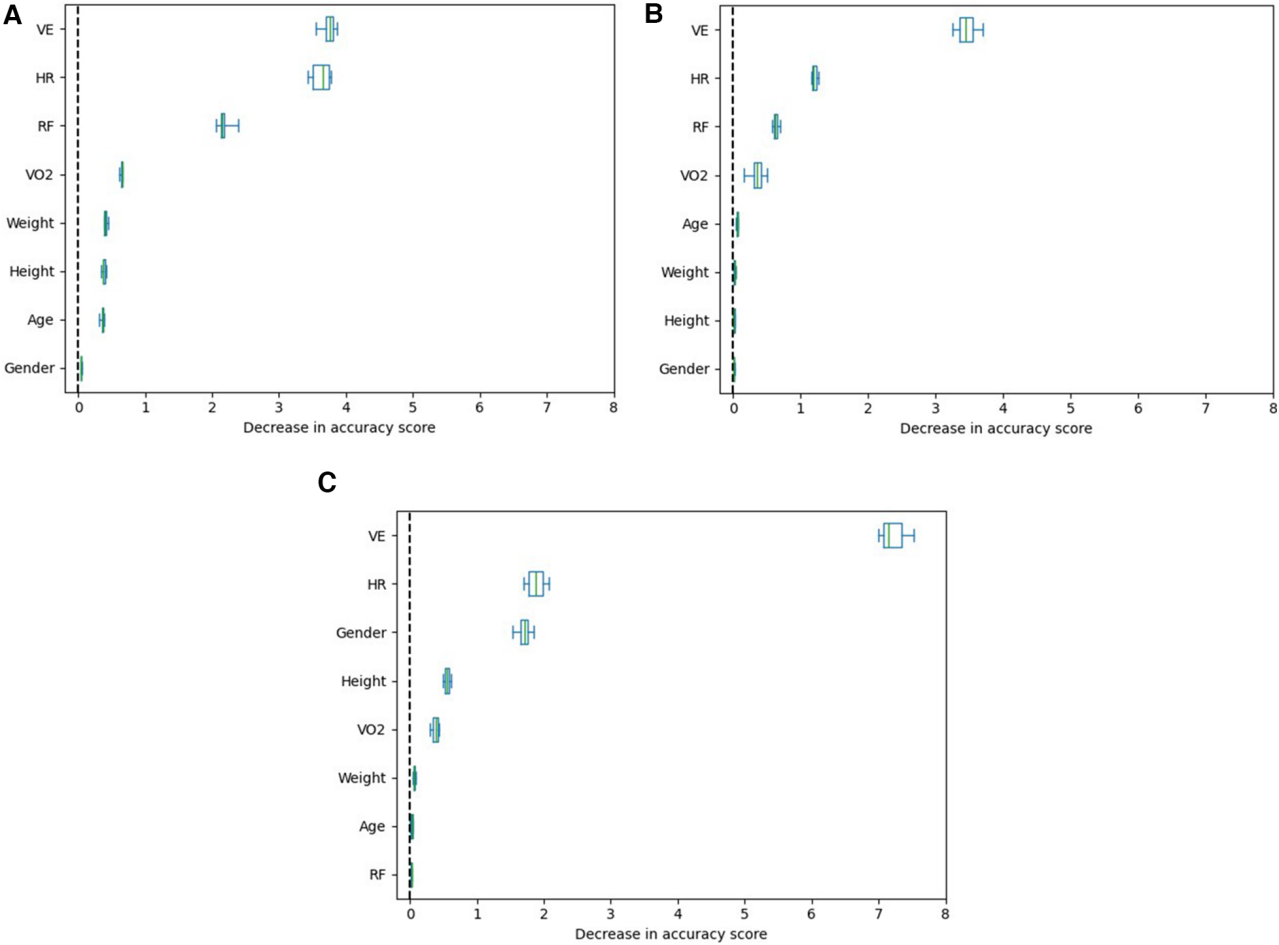
Permutation feature importance Boxplots for the three tested models. VE has been identified as the most important when estimating RPE, followed by HR, consistently across all three models. **(A)** RFR model feature importance, **(B)** MLP model feature importance, **(C)** Linear model feature importance.

PFI was used for each model to estimate the impact of individual input features for estimating RPE. PFI was repeated 10 times for each model to minimize bias. The output of a PFI calculation is the decrease in accuracy score of the model when each feature is randomized. Figure 2 shows the detailed results of the PFI calculations for each model. For all three models, VE was identified as the most important feature when estimating RPE (with median PFI scores of 3.8 for the RFR model, 3.4 for the MLP model, and 7.2 for the linear model), followed by heart rate (with median PFI scores of 3.7 for the RFR model, 1.2 for the MLP model, and 1.9 for the linear model).

## 4 Discussion

There is a growing interest in capturing perceived exertion during physical activity as a way to optimize training and potentially prevent injury due to fatigue. Recent studies already highlighted the importance of capturing respiratory measurements in sports. This study aimed to further investigate the impact of respiratory measures when estimating RPE using machine learning methods. Heart rate is one of the most commonly captured physiological metrics in sports, and is often used as a feature for modelling RPE in the current literature (5, 9–12, 14). A previous study found that both HR and respiratory measures can be used as predictors for RPE (13). As such, this study evaluated the importance of respiratory measures when modelling RPE alongside heart rate.

In this study, three regression models were evaluated. The RFR and the MLP models performed better than the linear models, which was expected due to their ability to capture more complex relationships. It was noted that during the cross-validation, the MLP exhibited signs of over-fitting for several participants, which was confirmed by allowing early stopping during the cross-validation. When early stopping is enabled, the MLP’s accuracy increases measurably, going from a RMSE of 1.882 and MAE of 1.511±1.123 to a RMSE of 1.668 and MAE of 1.299±1.046. The early stopping method is, however, not feasible when training the model with all available data, as early stopping requires a dedicated validation set. This shows that the performance of the MLP model is limited by the cohort size of this study, and has the potential to achieve much higher accuracy with more data available. With a larger dataset, it is also possible to further refine the hyperparameters of the MLP, which can potentially further improve the accuracy of the MLP.

The Permutation Feature Importance (PFI) calculations suggested that minute ventilation was the most impactful feature when modelling RPE across all models. Heart rate was found to be the second most important feature across these models. It was also shown that VO2 was of less importance when its margins were compared to VE and HR (VO2 PFI score median, RFR: 0.660, MLP: 0.354, LR: 0.383). This finding seems to be in line with earlier findings reported by de Almeida e Bueno et al. (13), who found minute ventilation to be better correlated (r=0.843) with RPE than heart rate (r=0.770), and both are better correlated with RPE than VO2 (r=0.705). These outcomes suggest that respiratory measures are crucial in understanding fatigue and warrant further investigations in sports settings. In order to capture perceived exertion, both respiratory and heart rate-based measurements should be taken into consideration.

While the collection of respiratory measurements in sports currently requires the use of expensive and obtrusive equipment, such as the COSMED K5 used in this paper, a number of recent studies have shown the possibility of collecting respiratory measures via more affordable and unobtrusive wearable systems, in the forms of chest straps (27, 28), instrumented garments (29), and instrumented mouthguards (13, 30). These systems also provide the opportunity to directly measure or estimate VE and Rf. While measuring VO2 on-field might be impossible without obtrusive devices capable of gas analysis, it is also import to note that the results of this study suggest that VO2 might be less relevant when estimating RPE using cardiorespiratory measures. When VO2 is omitted from the models presented in this paper, they achieve a similar level of accuracy to when VO2 is included as a feature. Without VO2 inclusion, the RFR model has an average RMSE of 1.794 and MAE of 1.430±1.082 (as opposed to a RMSE of 1.849 and MAE of 1.461±1.133 when VO2 is also considered), the MLP model has an average RMSE of 1.956 and MAE of 1.551±1.193 (as opposed to a RMSE of 1.882 and MAE of 1.511±1.123), and the LR model has an average RMSE of 2.173 and MAE of 1.734±1.309 (compared to a RMSE of 2.263 and MAE of 1.798±1.374). These findings support the future possibility of on-field capturing of athletes’ exertion levels using real-time cardiorespiratory sensing.

### 4.1 Limitations

Experimental conditions were kept as consistent as possible, whilst allowing for real-world representation. All measurements were taken outdoors at the same location. The air temperature and relative humidity were captured by the COSMED on the days of the experiments. No corrections for weather conditions were made in order to increase the generalisability of the results. The average peak ambient temperature across all tests was $22.85\pm 6.06^{\circ }$.

While the sensors used in this study have been shown to be valid with good accuracy in the literature, they are still prone to errors that can arise from poor contact due to motion. In the COSMED data, we detected spikes in the signal that are likely due to movements of the face mask. Furthermore, the HR data contained null values throughout the data, possibly due to temporary loss of adequate skin contact resulting from motion. The extent of the impact of these errors on the machine learning models should be further investigated in future studies as motion artifacts are inevitable in sports scenarios.

Only 80% of the volunteers completed two rounds, either due to availability conflicts or equipment failure, and around 3% of the data had to be omitted due to motion artifacts. Participants also differed in their athletic abilities, as shown by the final level reached on the Yo-Yo test. The leave-one-subject-out cross-validation was chosen, to minimize any bias due to these differences. Furthermore, the MLP model might be able to reach higher performance if more data were presented. Future studies should focus on expanding the dataset to capture more data for a wider range of participants, in order to better facilitate models that require large datasets. Lastly, our findings are based on a specific running interval test and the external validity might not be present for other forms of exercise.

Lastly, the cohort size can be considered small. However, it should be noted that it was very similar in size to previous published studies that are relevant for this topic Chowdhury et al. (11) (n=22); Rossi et al. (15) (n=22); de Almeida e Bueno et al. (13) (n=8); Albert et al. (12) (n=16). The limited cohort size would likely negatively impact the generalizability of this work, for this reason, and the reasons highlighted in the discussion above, we strongly recommend future studies to consider a larger cohort.

### 4.2 Conclusion

In this study, we investigated the feature importance when estimating RPE from cardiorespiratory measurements using machine learning methods. We compared three different regression models, linear regression, random forest regression (RFR), and multi-layer perceptron (MLP), and found that the RFR model had the best performance overall, with an average mean absolute error of 1.407±1.091 in a leave-one-subject-out cross-validation. The feature importance of each machine learning model was investigated using permutation feature importance, and we found minute ventilation (VE) to be the most important feature, followed by heart rate, when estimating RPE using cardiorespiratory signals. Future works that aim to estimate RPE using wearable sensors should therefore strongly consider including respiratory data.

## Data Availability

The raw data supporting the conclusions of this article will be made available by the authors, without undue reservation.
